# Effects of Exercise on Functional Recovery in Patients with Bipolar Depression: A Study Protocol for a Randomized Controlled Trial

**DOI:** 10.3390/metabo13090981

**Published:** 2023-08-30

**Authors:** Fumito Hamada, Hikaru Hori, Hitoshi Iida, Hiroyuki Yokoyama, Hiroko Sugawara, Akito Hatanaka, Leo Gotoh, Muneaki Ogata, Hiroki Kumagai, Rika Yano, Yuko Tomiyama, Tetsuya Yoshida, Yoshimi Yamaguchi, Ryo Asada, Masato Masuda, Yuta Okamoto, Hiroaki Kawasaki

**Affiliations:** 1 Department of Psychiatry, Faculty of Medicine, Fukuoka University, Fukuoka 8140180, Japan; fukuoka2310@gmail.com (F.H.); hitoshi.628@gmail.com (H.I.); hiroko-sugawara@umin.ac.jp (H.S.); gleo@fukuoka-u.ac.jp (L.G.); ky_8r7t@yahoo.co.jp (R.A.);; 2 Laboratory of Neuroscience, Department of Psychiatry, Faculty of Medicine, Fukuoka University, Fukuoka 8140180, Japan

**Keywords:** bipolar disorder, depression, exercise, randomized controlled trial, cognitive function, recovery, biological marker

## Abstract

Treatment of bipolar disorder is prone to prolongation despite various treatments, including medication. The efficacy of exercise treatment (i.e., interventions involving physical exercise and sports intervention) for major depressive disorders has been reported for depressive symptoms, cognitive function, and sleep disturbances. However, its efficacy for bipolar disorder has yet to be established. We designed a randomized, controlled, double-blind clinical trial that includes 100 patients with bipolar disorder aged 20–65 years. This will be a cluster-randomized, two-group trial that will be conducted in ten psychiatric hospitals. The hospitals will be randomly assigned to an exercise intervention + treatment as usual (exercise) group or a placebo exercise intervention (stretching) + treatment as usual (control) group. Patients will be assessed using an extensive battery of clinical tests, physical parameters, sleep status, biological parameters (cytokines, neurotrophic factors), and genetic parameters (DNA and RNA) at baseline after a 6-week intervention period, at 10-week follow-up, and at 6-month follow-up. This innovative study may provide important evidence for the effectiveness of exercise in the treatment of bipolar depression based on clinical, biological, genetic, and physiological markers.

## 1. Introduction

Bipolar disorder (BD) is a mood disorder affecting approximately 400 million individuals worldwide [1]. Moreover, BD ranks as one of the top 20 leading causes of disability among all diseases worldwide [2]. BD is a chronic disorder characterized by recurrent manic and depressive episodes. It causes mood swings, increased suicide rates [3,4], self-injuries and other harms [5,6], cognitive impairments [7,8], sleep–wake rhythm disturbances [9], impaired social functioning [10,11], and presenteeism and absenteeism [12]. BD is treated with a combination of therapies, including medications, such as mood stabilizers and atypical antipsychotics, psychotherapy, family education, and social skills training. However, even with these treatments, it is difficult to maintain remission, and issues, such as repeated relapses, exist. In addition, many patients with BD are depressed for a long time, and several cases of prolonged depression have been reported [13,14]. Manic states quickly improve with the concomitant use of mood stabilizers and antipsychotics; however, improvement of depressive states takes a long time. Most guidelines do not recommend antidepressant use for patients with BD and state that antidepressants may be considered only for a limited number of patients. Despite these guidelines, antidepressants are still used in several clinical situations [15,16,17]. This is due to poor treatment results, improvements in BD depressive episodes, and the lack of various treatment options.

Meta-analyses have reported various effects of exercise treatment (i.e., interventions involving physical exercise and sports intervention), such as prevention and treatment of major depressive disorder (MDD), improvement of cognitive function [18], and improvement of sleep [19]. Previous studies that focused on the effects of exercise in patients with bipolar disorder have used periods ranging from 10 days to 32 weeks. Several 6-week studies have been reported, which we believe is an appropriate intervention treatment period [20]. On the other hand, few studies have examined the effectiveness of exercise therapy in BD. In recent years, the treatment of psychiatric disorders has been expected to improve not only mental symptoms but also social functioning. In particular, the idea of emphasizing recovery in the treatment of psychiatric disorders is gaining popularity worldwide. In various clinical trials, it has been pointed out that it is desirable to set social and cognitive functions as primary outcomes. Metabolomic studies have reported that diverse BD metabolites are associated with changes in biochemical pathways, such as mitochondrial/energy metabolism, oxidative stress, amino acid metabolism, and lipid metabolism. Previously, we have reported the results of studies on catecholamine metabolites, brain-derived neurotrophic factor (BDNF), and cytokine levels [21,22,23,24]. Several studies have reported a decrease in BDNF in patients with BD [25,26]. Exercise interventions increase the levels of neurotransmitters and neurotrophins, such as cortisol, β-endorphin, and BDNF [27,28]. BDNF may also increase neuronal adenosine triphosphate production [29]. BDNF also plays an important role in brain plasticity, neuronal difference and survival, neuronal function, and neurogenesis stimulation by activating tropomyosin receptor kinase B (TrkB). There are two forms of BDNF receptors [30]: active TrkB-FL and inactive TrkB-T1. Exercise increases the levels of neurotransmitters and neurotrophins, such as cortisol, β-endorphin, and BDNF, via the peroxisome proliferator-activated receptor-γ coactivator-1α/fibronectin type III domain-containing 5 pathway [31]. Thus, the BDNF signaling pathway may be involved in BD pathophysiology.

Moreover, patients with BD have a higher incidence of metabolic syndrome, hyperlipidemia, weight gain, and hypertension than the general healthy population [32,33,34,35]. Some bipolar medications affect weight gain and the metabolic system; however, a previous study reported a high prevalence of metabolic syndrome in drug-naïve patients with BD [36]. Among the several loci identified in large-scale genome-wide association studies of BD, the fatty acid desaturase 1/2 (FADS1/2) region was first detected in a Japanese population [37] and replicated in a European population [38,39]. Furthermore, FADS1/2 mutant mice showed BD-like episodic behavioral changes, and both supplementation with unsaturated fatty acids and lithium administration prevented these behavioral changes [40]. These findings suggest that lipid metabolism is involved in the pathogenesis and treatment of BD [41]. Moreover, a pro-inflammatory state exists in BD and its comorbidities [42], and a recent systematic review on the applications of Mendelian randomization suggested a causal implication of pro-inflammatory status (and C-reactive protein) in the risk of developing BD [43,44]. This pro-inflammatory state affects brain networks [45,46], particularly hippocampal neurogenesis [47]. Physical exercise reduces inflammation and stress, enhances hippocampal neurogenesis [28], and disrupts hippocampal connectivity in patients with BD [48]. Thus, stress, inflammation, and hippocampal neurogenesis may be promising candidates for providing the mechanistic grounds for our hypothesis.

Owing to such a high risk of physical problems, monitoring and interventions for physical health conditions among patients with BD were suggested by a recent commission [49]. Exercise training enhances insulin sensitivity and glucose metabolism in the skeletal muscle [50], and the elevation in energy expenditure associated with repeated training directly influences adipocyte metabolism [51]. Therefore, exercise therapy is likely to positively affect the physical health of patients with BD.

In recent years, the goal of treatment of psychiatric disorders, including BD, is not improvement of psychiatric symptoms or clinical recovery, but functional and personal recovery. Service users with mental health problems have highlighted the importance of measuring change beyond symptoms. However, many previous studies have used improvement in clinical symptoms as the primary outcome and have not studied recovery and other functional aspects of recovery as the primary outcome. In our current study, we decided to use a scale that can assess recovery as the primary outcome. At the same time, psychiatric symptoms, cognitive function, sleep–wake rhythms, and biomarkers should be evaluated simultaneously to examine recovery in more detail.

We posit that a regimen of physical exertion augments the capacity of patients and that this elevation manifests itself in their cognitive, bodily, biological, and physiological metrics. Therefore, this study aims to determine whether exercise therapy as an adjunct therapy to pharmacotherapy for BD improves the level of psychosocial functioning in patients with BD. The secondary aim will be to determine the effects of exercise on depressive symptoms, sleep, quality of life, cognitive function, personal recovery and biological variables.

## 2. Materials and Methods

### 2.1. Study Design and Flowchart (Figure 1)

This study will be named the CERF-BD (Clinical Exercise Research in Fukuoka University in Bipolar Disorder). This will be a multicenter, matched-pair cluster-randomized controlled trial that will be conducted in 10 psychiatric institutions (one university hospital (Fukuoka University Hospital) and nine psychiatric hospitals (Aburayama Hospital, Amagi Hospital, Fukuma Hospital, Gannosu Hospital, Mito Hospital, Niji to Umino Hospital, Shiranui Hospital, Shiroishi Hoyoin, and Wakahisa Hospital) addressing inpatients diagnosed with BD depressive episodes according to the Diagnostic and Statistical Manual of Mental Disorders, Fifth Edition (DSM-5) criteria [52]. The institutions will be randomly assigned to either an exercise group (exercise intervention + treatment as usual (TAU)) or a control group (stretching (ST) + TAU). If there is a request for additional research sites, they will be added as research participating sites and allocated as appropriate.

**Figure 1:**
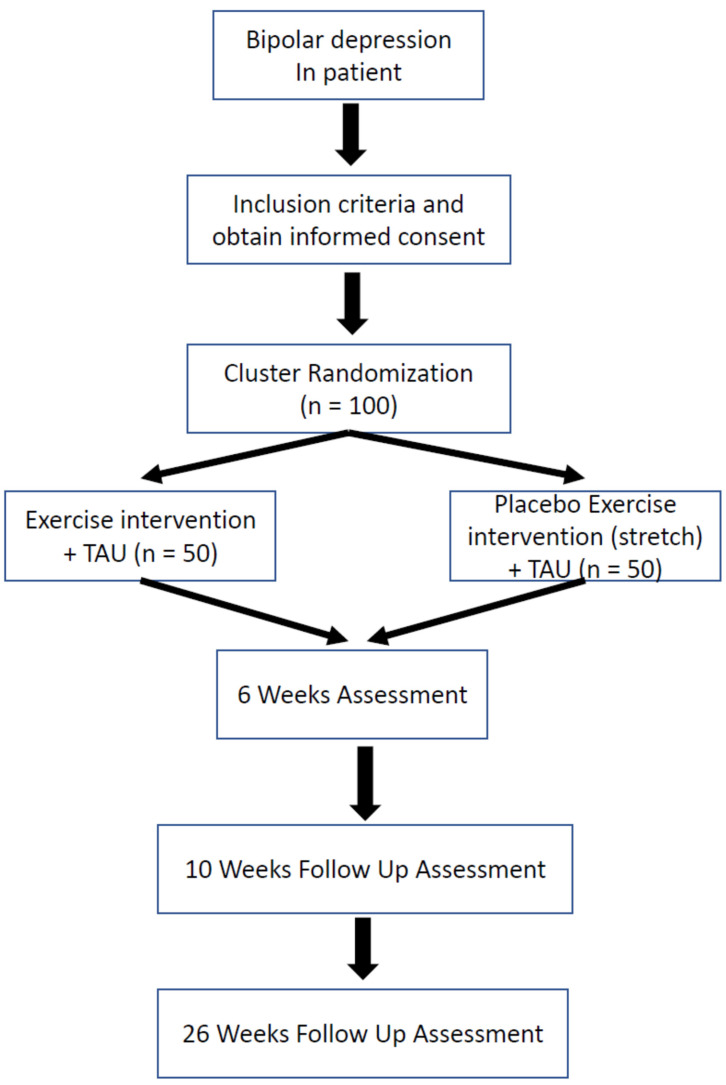
Study protocol. TAU: Treatment as usual.

TAU will involve continuing the usual pharmacotherapy for BD and psychotherapy once or twice a week. As this study aims to examine the effects of exercise therapy, no change in medication will be allowed during study participation. Patients will drop out of this study when their psychiatric symptoms change sufficiently to require a change in medication. We will also conduct a survey at the end of the study in which all the subjects will be asked to indicate which group they believe they were assigned to.

### 2.2. Participants

#### Patients

The assemblage of patients with BD will be procured by adept and accomplished psychiatrists from the inpatient milieu of the research institute.

The inclusion criteria will be as follows:

- Diagnosis of BD according to the DSM-5 [52];
- Current depressive episode;
- A total of 6–15 points on the Quick Inventory of Depressive Symptomatology-Self-Report (QIDS-SR);
- Age between 20 and 65 years.

The exclusion criteria will be as follows:

- Intellectual disability (assessed based on the DSM-5);
- History of cranial trauma with loss of consciousness;
- Physical diseases that cause mental health problems;
- Pervasive developmental disorders;
- Pregnancy or breastfeeding;
- Severe or uncontrolled cardiovascular risk factors, such as unstable coronary artery disease, uncontrolled hypertension, malignant ventricular arrhythmia, atrial fibrillation, exercise-induced ischemia, and ventricular failure;
- Other significant medical conditions, including, but not limited to, chronic or recurrent respiratory, gastrointestinal, neuromuscular, or musculoskeletal problems that interfere with exercise;
- Inflammatory diseases;
- Clinical variables.

### 2.3. Recruitment

Potential participants will be primarily inpatients at each of the study facilities. Postings advertising the study will be made in outpatient psychiatry departments, and patients interested in the study can contact us and inquire about information and participation. Recruitment is also widely advertised on our website and on X (Twitter) regarding the study. Prospective participants can be screened by contacting the researchers.

### 2.4. Sociodemographic Variables

Data on sociodemographic variables and BD-related variables (sex, age, education, family history of psychiatric disorders, number of mood episodes, medication history, employment status, marital status, number of hospitalizations, and exercise habits) will be collected at baseline.

### 2.5. Psychosocial Functioning

The competency of patients will be assessed using the Functioning Assessment Short Test (FAST) [53]. This tool is a succinct means of evaluating functional impairment in patients with psychiatric disorders. It encompasses 24 facets spanning six distinct domains of competency: self-sufficiency, professional proficiency, cognitive ability, fiscal affairs, social interactions, and leisure pursuits. Each facet is rated on a scale of 0–3 points (0, no difficulty; 1, mild difficulty; 2, moderate difficulty; 3, severe difficulty), yielding a cumulative score ranging from 0 to 72 points. The CERF-BD study’s primary outcome is change in FAST scores; the FAST rater will be an experienced psychiatrist independent of the study. The FAST evaluation will be conducted by telephone or in an online interview (off camera). This will allow the rater to be blind to which group the study participants are assigned. In addition, it will be meaningful because study participants will not know which evaluation period they are in.

### 2.6. Depressive Symptoms

The severity of depressive symptoms will be measured using the QIDS-SR [54], which consists of 16 questions assessing the severity of nine DSM-5 diagnostic symptoms of depression. The total severity score ranges from 0 to 27. We will also use the 17-item Hamilton Rating Scale for Depression to evaluate depressive symptoms.

### 2.7. Neurocognition

THINC-it^®^ [55] is a newly developed digital screening instrument for cognitive dysfunction in patients with MDD. The following four objective cognitive tests are included in THINC-it^®^: choice reaction time, n-back, digit symbol substitution, and Trail-Making Test-B, as well as subjective cognitive impairment (Perceived Deficits Questionnaire-Depression 5-items).

Spotter: The Spotter test is used to assess attention and response time. In this test, participants are instructed to respond by pressing keys on the left- or right-hand side of the keyboard corresponding to the direction to which an arrow on the screen is pointing. The test consists of 40 tasks and evaluates the percentage of correct answers and mean reaction time.

Symbol Check: The Symbol Check task measures working memory (one-back task) and attention skills. The participant is presented with a series of stimuli at a constant rate. The task is to map the currently presented stimulus on arrow keys of a keyboard to one they have recently seen in the stream. The test consists of 40 tasks and evaluates the percentage of correct answers and mean reaction time.

Codebreaker: The codebreaker test, which is modelled on the digit symbol substitution test (DSST), measures attention, perceptual speed, motor speed, visual scanning, and memory. It requires the examinee to transcribe a unique geometric symbol with its corresponding number. The examinee is initially shown a key containing the numbers from 1 to 6. Under each number, there is a corresponding geometric symbol. The participant is then shown a series of numbers on the screen and asked to match the number with the corresponding geometric symbol. The test evaluates the total correct number and average completion time.

Trails: The Trails test is designed to test elements of executive function. This task is based on Trail-Making Test-B. Participants are instructed to connect a set of 18 dots as fast as possible while still maintaining accuracy. In this task, the participants alternate between the smallest number and the hiragana. It can provide information about visual search speed, scanning, speed of processing, mental flexibility, as well as executive functioning. The test evaluates the total contacts, total time, total wrong nodes, and total errors.

### 2.8. Quality of Life

The World Health Organization Quality of Life Assessment (WHO-QOL-26) is the Japanese version of the WHO Quality of Life-Brief Version translated and tested for equivalence by Tazaki and Nakane [56]. The WHO-QOL-26 comprehensively measures subjective well-being and comprises 24 items in four domains: physical health, psychological health, social relationships, and environment, and two items for overall QOL and general health. Each item (e.g., “To what extent do you feel that physical pain prevents you from doing what you need to do?”) is rated on a 5-point Likert scale ranging from 1 (not at all/very dissatisfied) to 5 (a lot/very satisfied). It demonstrates adequate reliability (Cronbach’s α = 0.66–0.84) and discriminant validity.

### 2.9. Personal Recovery

The Questionnaire about the Process of Recovery (QPR-J) will be used to measure personal recovery. The QPR is a 22-item, consumer-rated questionnaire used to assess personal recovery; each item is rated on a 5-point Likert scale that ranges from 0 (disagree strongly) to 4 (agree strongly), with higher scores indicating increased recovery [57].

### 2.10. ActiGraph^®^

Objective sleep and physical activity will be measured using an ActiGraph^®^ (ActiSleep-BT Monitor; ActiGraph Inc., Tokyo, Japan) worn on the nondominant arm for 7 days before each assessment time point (i.e., 7 days prior to study entry and 7 days prior to the 6-week assessment point). The participants will be instructed to wear the accelerometer, attached to the wrist by an adjustable belt, on all days, except during bath time.

Sleep parameters, such as total sleep time, sleep efficiency, sleep onset latency, and wake after sleep onset, will be calculated using ActiLife^®^ (ActiGraph, Inc., Tokyo, Japan). A validation study of this device showed moderate-to-high agreement with sleep parameters as measured by polysomnography [58].

### 2.11. Biological Variables

Blood sampling will be performed at baseline and week 6 (end of the intervention). Venous blood specimens (7 mL) will be procured by nursing personnel between 6:00 and 9:00, following a nocturnal fast, in polypropylene tubes containing ethylenediamine tetraacetic acid. The samples will be maintained at a temperature of 4 °C until subjected to analysis. These samples will enable the analyses of the association between biomarkers and psychosocial functioning and the effects of the intervention on biomarker levels. Peripheral blood will be collected at each time point of assessment to obtain DNA, RNA, and plasma for later biomarker analysis (e.g., genetics, gene expression, sequencing, proteomics, epigenomics, serum/plasma levels of biomarkers). Whole blood in the collection tube will be gently stirred until uniform, and 600 μL will be transferred for DNA and RNA extraction. The remaining blood will be centrifuged (2500× *g* × 20 min, 18 °C) and used to collect plasma samples. In total, 900 μL of the plasma will be allocated for the extraction of cell-free RNA, and the remaining plasma will be used for the measurement of proteins and lipids. Whole-blood DNA, whole-blood RNA, and plasma cell-free RNA will be extracted using an extraction kit according to the manufacturer’s protocol. The concentrations of these samples will be measured using a spectrophotometer (nano200) and fluorometer (Qubit2.0). The samples will be stored in secure refrigerators, all of which will be monitored by a central alert system 24 h/day for 7 days/week at Fukuoka University.

Clinical Trial Registration: UMIN000045877.

### 2.12. Exercise vs. Stretching Groups

All participants in this study will perform exercise or ST during inpatient treatment. Both groups will receive exercise or ST for approximately 40 min thrice a week for 6 weeks.

#### 2.12.1. Exercise Group

The exercise intervention will be provided by at least two trained medical staff, including a mental health exercise instructor certified by the Japanese Association of Sports Psychiatry, based on the National Institute for Health and Care Excellence (NICE) depression guidelines in the UK and the WellCare exercise program (aerobic + resistance exercise). Exercise intensity will be based on 50–76% of the maximum heart rate (220–age) to ensure mild-to-moderate intensity. Subjective exercise intensity during exercise will be confirmed using the Borg scale [59].

#### 2.12.2. Stretching Group

ST will consist of 40 min of ST exercises. Multiple muscle conglomerates, including the thighs, shanks, gluteals, shoulders, and back, will be stretched at equivalent intervals between each set of stretches. Exercise intensity in the ST group will be based on 50% or less of the maximum heart rate (220-age) to be mild. The training sessions will be conducted in a hospital room restricted to these activities and supervised by the research members.

### 2.13. Procedures

Consent for the study will be obtained from the subjects by checking Inclusion and Exclusion criteria in hospitalized bipolar patients; patients who consent to the CERF-BD study will be fitted with an ActiGraph^®^ 1 week prior to study entry (day 7). All outcomes will be assessed at baseline (day 0). The primary endpoint, the FAST score, will be assessed in a blinded fashion. Exercise or ST will be performed 6 weeks (day 42) after the baseline assessment. Exercise will be performed 3 times per week. Basically, the day of the week will be fixed for the intervention, but the day of the week can be changed if there are special reasons such as national holidays (e.g., New Year’s Day). In addition, if the study participant, researcher, or attending physician deemed it difficult to participate in the exercise therapy due to coronavirus infection or other physical illness or mental condition, the participant was considered absent. Dropout was defined as an exercise therapy compliance rate of 80% or less. BD primary medications (mood stabilizers, antipsychotics, and antidepressants) were not changed or adjusted during the study period. At the end of the study, the same tests as at baseline will be performed. Two follow-up assessments will be performed (days 70 and 182). The study schedule is shown in Table 1.

### 2.14. Statistical Analyses

Statistical analyses will be performed using the Statistical Package for the Social Sciences software, and a *p*-value < 0.05 will be considered statistically significant. The normality of quantitative variables will be assessed using the Kolmogorov–Smirnov test, which is a statistical test used to determine if a sample of data comes from a population with a normal distribution. If the test indicates that the data are normally distributed, the results will be expressed as mean and standard deviation (SD), which are commonly used measures of central tendency and variability.

The FAST score at 6 weeks will be used as the primary outcome indicator. Differences between groups in FAST scores will be compared first using the chi-square Levene test, and if the variances are chi-square, a *t* test will be used; if the variances are not chi-square, differences between groups in FAST scores will be compared using a paired *t* test. Analysis of covariance will be used to control for baseline confounders and to further explore the differences in scores between the two groups before and after the intervention. Six-week scores on other scales and biological indicators will be used as secondary outcomes, using similar methods as described above. Missing data will be subjected to the multiple fill method, and sensitivity analysis will be performed before and after filling. An alpha = 0.05 will be used. Intentional analysis (ITT), consistent with scenario set analysis (PP) as a sensitivity analysis, will mainly be used in this study.

In our randomized controlled trials, we specify multiple outcomes. For this reason, we perform multiple comparison corrections to properly control the familywise error rate.

### 2.15. Sample Size

Sample sizes will be calculated to determine clinically meaningful differences. In a study examining FAST improvement in the primary outcome [60], the mean difference between the two FAST groups was set to 5 and the SD to 10. The design effect was considered for the calculation of the cluster sample size, the ICC was set to 0.02, and a dropout rate of 20% was considered. The total number of patients in each group was set to 100, as there were 49.2 patients in each group.

### 2.16. Ethical Consideration

This study has been approved by the Clinical Research and Ethics Centre of Fukuoka University. Written informed consent will be obtained from all participants before enrollment in this study. This study is registered with the UMIN Clinical Trials Registry (UMIN000045877).

## 3. Discussion

The main purpose of this study is to determine whether exercise intervention for BD improves psychosocial functioning. Although psychiatric studies have focused on symptom improvement, the treatment goal in recent years has been functional recovery, including improvement in psychosocial functioning [61]. The results of this randomized controlled trial will help us understand whether exercise intervention for BD improves psychosocial functioning. We will not only be able to prove the effects of exercise on psychosocial functioning in patients with BD, but also learn about a wide range of other effects, including on depressive symptoms, sleep, quality of life, cognitive function, personal recovery, and biological variables. The strength of this study is that it simultaneously measures multiple biomarkers. We hope that this will clarify the effects of exercise on metabolism, allow us to correlate metabolism with BD, and predict treatment responses to exercise therapy.

Exercise therapy is effective in preventing MDD [62,63], reducing symptoms [64], improving cognitive function [18], and improving sleep [19]. Several studies have examined the biological markers of the effects of exercise treatment on MDD [65,66,67,68]. However, only a few studies have examined exercise interventions for BD. To the best of our knowledge, no randomized controlled trials on the efficacy of exercise interventions for BD have been conducted. Moreover, BD guidelines hardly mention the efficacy of exercise.

In this study, we will examine the effect of adding exercise therapy to pharmacotherapy for BD improvement in terms of psychosocial functioning, depressive symptoms, physical functioning, cognitive functioning, sleep–wake rhythm, and biological aspects. To the best of our knowledge, no previous study has investigated the effects of exercise therapy on psychosocial functioning, psychiatric symptoms, cognitive functioning, physical functioning, sleep–wake rhythms, biological markers, and genes in patients with BD. Through this study, it will be possible to learn about the effectiveness of exercise therapy for BD and elucidate its mechanisms. In particular, this study will evaluate genes (e.g., FADS), inflammatory cytokines, and BDNF, which will be useful for predicting mechanisms and therapeutic responses.

This study has multiple strengths. First, the primary endpoint is the FAST score to measure psychosocial functioning. In recent years, the treatment goal for BD has often been set at recovery. The FAST is said to be appropriate for assessing recovery. Furthermore, the evaluators of the FAST will be blinded to which group the subject is in. This is one of the unique features of this study. Second, we set up a stretching group as the active control group. Exercise therapy studies often include a waitlist group, and some have pointed out that this is a problematic approach. In a randomized controlled trial using a waitlist group, subjects would know whether they were assigned to the exercise group or the waitlist. In pharmacotherapy studies, the placebo group is also likely to have a placebo effect because of the behavior of taking the placebo internally. We will also conduct a survey at the end of the study in which all the subjects will be asked to indicate which group they believe they were assigned to. This will allow us to understand how effective the randomization was, as well as to evaluate the possibility of underestimating the effect of exercise therapy, as noted in the limitations below. Third, we will be conducting a variety of evaluations, including of biological markers, metabolic markers, inflammatory markers, and neurotrophic factors, while conducting a randomized study. This will likely lead to advancements in personalized medicine.

This study has some limitations; for example, participants cannot be followed up owing to recurrence and withdrawal of consent, but all patients are inpatients and can be medically treated immediately. This study may not be generalizable as it will be conducted on Japanese patients with BD. Future studies should examine the duration of the exercise therapy. In addition, because this is a randomized controlled trial conducted in a multicenter study, the possibility that there are differences in the background of BD at each institution cannot be ruled out. In this study, an ST group will be established as an active control group. This may reduce the effectiveness of the intervention. In addition, the inflammatory state is highly variable; therefore, we need to properly understand and manage the patient’s physical condition.

## 4. Conclusions

This study will provide important evidence of the effectiveness of exercise therapy in the treatment of BD. The use of recovery assessment as the primary outcome, a placebo exercise therapy group as the control group, and the evaluation of biological, physiological, and genetic aspects will allow for a multifaceted analysis approach. In particular, by collecting biological markers, it will be possible to elucidate the mechanism of the improvement of depression and propose personalized medicine. This will provide the first study results on the effectiveness of exercise therapy for BD.

## Figures and Tables

**Table 1:** Schedule of assessments.

	Screening	0 w	4 w	6 w	10 w	26 w
Bipolarity index	O					
FAST		O		O	O	O
QIDS-SR, HAM-D	O	O	O	O	O	O
THINC-it^®^		O		O	O	O
Blood sampling (general blood sampling, genes, and biomarkers)		O		O		O
Physical measurements (height, weight)		O		O		O
WHO-QOL (quality-of-life assessment)		O		O	O	O
Personal recovery scale (condition assessment)		O		O	O	O
Physical checkup (grip strength test, repetitive side jumping)		O		O		O

FAST, Functioning Assessment Short Test; QIDS-SR, Quick Inventory of Depressive Symptomatology-Self-Report; HAM-D, Hamilton Rating Scale for Depression; WHO-QOL, World Health Organization Quality of Life Assessment.

## Data Availability

No new data were created or analyzed in this study. Data sharing is not applicable to this article.
